# "The Hospital" Nursing Mirror

**Published:** 1898-02-19

**Authors:** 


"The Hospital" Nursing Mirror.

Being the Nursing Section of "The Hospital."

[Contributions for this Section of "The Hospital" should be addressed to the Editor, The Hospital, 28 & 29, Southampton Street, Strand, London, W.C., and should have the word "Nursing" plainly written in left-hand top corner of the envelope.]

### News from the Nursing World.

### THE QUEEN AT NETLEY.

The Queen's visit to her wounded soldiers at Netley gave the greatest pleasure to all in the hospital. Favoured by fine weather, Her Majesty crossed from Osborne to Portsmouth in the royal yacht *Alberta*, travelled thence to Netley by special train, and drove to the hospital. The Queen then proceeded in her wheeled chair on a tour throughout the wards, and spent a considerable time in conversing with the suffering men, leaving after a stay of about an hour. Her Majesty, whom we are sorry to say appeared delicate, was greeted with the warmest enthusiasm, not only by the soldiers whom she went to see, but by the crowds at the way side. Several presentations were made to Her Majesty. Amongst those so honoured was Miss Norman, the matron, who afterwards accompanied the Queen through the wards. Her Majesty has announced her intention of repeating her visit upon her return from the continent. She has also asked that photographs of the patients she saw may be sent to her, with particulars of their cases noted on the back.

### A ROYAL REGISTER.

Amongst the books which the Queen delights to read is the register of the marriages and christenings of the Royal family, now in the care of the Rev. Edgar Sheppard, Sub-Dean of the Chapel Royal. The first entry is that of the Queen's own marriage, and then come in due order those of her children, grandchildren, and great-grandchildren, no matter where the ceremonies may have taken place. This volume, apart from the interest attaching to it as the record of our Royal house, is valuable as containing the autographs of nearly all the sovereigns of Europe. The signatures of notable folk, for instance, at the marriage of the Duke and Duchess of York, occupy four pages.

### NURSES' CERTIFICATES UNDER THE POOR LAW.

Some time ago we drew attention to the action of a nurse who possessed a two years' certificate arranging to return to her training-school as sister after having made an agreement with the authorities thereof to have her three-year certificate granted her at the expiration of the period that must elapse before she could complete her three years' service from the date of her first entry. Doubts were raised as to whether the Local Government Board would recognise this certificate for the post of superintendent nurse, and we interrogated them upon the matter. The following letter which we have received in reply is valuable: "Gentlemen,—I am directed by the Local Government Board to advert to your communication of the 15th ultimo, and with reference to the inquiry therein contained, to inform you that, without expressing any opinion as to the particular case mentioned therein, it may be stated generally that there does not appear to be any reason why a nurse who has left the institution at which her training was commenced should not return to it to complete it."

### DEATH OF THE LADY SUPERINTENDENT OF THE BOMBAY PLAGUE HOSPITAL.

We record with deep regret the death of Miss Frances Eva Morgan, lady superintendent of the General Plague Hospital and Nursing Establishment, Bombay. Miss Morgan, who was only thirty-one years of age, was trained at St. George's Hospital, and was sent out to India in October last. The India Office has received an official telegram announcing that her death took place at Poona on the 13th inst. Volunteer nurses must therefore remember that when they go out on plague duty they literally carry their lives in their hands, as Miss Morgan's death only too surely proves. The call for English nurses to fight the plague has been so freely responded to that, although twenty-five nurses left a week or two ago, there are still about forty on the reserved list awaiting selection. We are glad to learn that, in spite of the sad loss here chronicled, the new plague encampment at Poona is in many respects an improvement on the old one. The jackals and snakes are not now seen, and Captain Lloyd Jones and the Lady Superintendent, Miss Wheatley, consider the comfort of the nurses under them very carefully.

### ROYAL NATIONAL PENSION FUND FOR NURSES.

We are glad to learn that the late Mr. W. H. Burns has left a legacy of £1,000 to the Nurses' Pension Fund. This, of course, will be devoted to augmenting the Donation Bonus Fund. After the devoted service rendered to the Fund during his lifetime by Mr. Burns, this further proof of interest and kindness is especially gratifying, and must increase the gratitude which the nurses of the Pension Fund entertain towards his memory.

### MISS LUMSDEN'S RETIREMENT.

Miss Rachel Lumsden, whose contemplated retirement we were able to announce some weeks ago, in connexion with the Queen's message to her, was the recipient of a hearty vote of thanks at the annual meeting of the infirmary on the 9th inst. It was as follows: "That the best thanks of the managers of the Royal Infirmary be accorded to Miss Frances Rachel Lumsden for her most valuable services to the institution as honorary superintendent for the long period of twelve years." The vote, which was proposed by Professor Dove Wilson, seconded by Mr. David Littlejohn, and supported by the Rev. C. C. Macdonald and the Lord Provost in highly eulogistic terms, was carried with applause.

### AN HONOURED NURSE.

In the despatches of Major-General A. G. Yeatman Bigg, C.B., quoted in the *London Gazette* for the 11th inst., appears the name of Miss Theresa McGrath. She receives honourable mention for valuable services rendered to the medical officers in the operations in the Samana Range in the Kurrani Valley during August and September last. The despatches speak of Miss McGrath's work as being enthusiastically appreciated by the men, an appreciation which their commander says is fully deserved. Miss McGrath, who has won this honourable distinction, it may be remembered, was nurse to the children of Major Des Voeux, but was attached to the hospital by the command of Major-General Yeatman Bigg.

### THE NURSES' CO-OPERATION SHOWS THE WAY.

At a meeting held at 24, Park Lane, by the kindness of the Hon. T. A. and Lady Idina Brassey, to discuss means of ameliorating the position of working-women, Miss Honnor Morten instanced the success of the Nurses' Co-operation as an example of the value of union, proving how it not only raises the earnings and status of the individual but of the profession to which they belong. The special scheme on foot is for dress-makers, it being hoped that a body of reliable work-women will be trained to fill a gap between the ruinously dear perfection of the shops and the slop work of the half-trained apprentices. No one will appreciate the benefits that such a society may confer more than nurses, who are by no means singular in liking their clothes well cut and trimly fitting.

### THE NURSES' NEEDLEWORK GUILD.

The Nurses' Needlework Guild is becoming popular, and the hon. secretary, Nurse Theobald (4, Bolsover Street, W.), is working diligently to make it successful. It is managed by a committee of nurses and some who are not nurses. These latter have been added as it is not always convenient for nurses to attend to business outside their professional duties. Twenty-three new members have joined this year, and any others wishing to add their mite to this useful work may, if nurses, become members; or, if not, associates.

### GLASGOW TRAINING HOME FOR NURSES.

The Marquis of Breadalbane presided at the annual meeting of the Glasgow Training Home for Nurses on the 9th inst. In addressing a distinguished assembly his Lordship said that there were 108 nurses on the staff, and that nurses from the home were known all over Scotland. The financial statement was not quite so satisfactory as it might be, owing partly to the fact that more nurses were entitled to the higher scale of remuneration, and partly because the celebration of Her Majesty's Diamond Jubilee had diverted subscriptions to other objects. He called attention to the fact that the institution that day had been in existence for 24 years, that it had been under the management of Miss McAlpin and her sister the whole of the time, and that it was impossible to speak too highly of the way they had conducted it. It is a matter of congratulation that weary and sick nurses on the staff have now the delightful prospect of a convalescent home soon to be opened at Busby through the liberality of the late Dr. Samuel Moore. His Lordship concluded his remarks by reading a letter of regret and appreciation from Lord Inverclyde, who was unable to be present.

### MUIR HALL.

A new residential hall for women medical students at Edinburgh has lately been built by a company. The management is in the hands of a committee, and Miss Robertson is resident superintendent. There is accommodation for 23 students. The arrangements for their comfort seem good, and the prices economical, namely, 16s. 6d. to 25s. a week. It is named Muir Hall, after the late Lady Muir, wife of the Principal of the University, and it was opened a short time ago by the Duchess of Buccleuch.

### THE MANCHESTER HOSPITAL WORK SOCIETY.

The aim of the Manchester Hospital Work Society is to make and supply the different hospitals and homes with suitable garments for the patients to wear in the wards. Last year 17 institutions were fortunate in having a share of the good things thus provided, which numbered 1,776. The Lord Mayor took the chair at the ninth annual meeting on January 24th, and Mr. Tom Jones read the report for his wife, who is the hon. secretary. The Pendlebury Children's Convalescent Hospital at St. Annes-on-the-Sea, and the Skin Disease Hospital, in Quay-street, have been added to the list of hospitals participating in the distribution.

### LONDON SPECTACLE MISSION.

The fifth annual report of the London Spectacle Mission is before us, and we see that very nearly eight hundred people have been helped by this society last year. It was founded and carried on at his own expense by Dr. E. J. Waring, C.I.E., until his death. It is now managed by a committee, and Miss Waring, 197, Sutherland Avenue, W., is the hon. secretary. Tickets are given to subscribers entitling them to recommend so many persons for spectacles. If, however, they have no one they wish to benefit, the hon. secretary will be happy to give the names of clergymen in poor parishes who are desirous of securing them for their poor parishioners.

### FURNISHING A NURSES' HOME.

Mrs. Albert Lord, who was Mayoress of Newcastle two years ago, set herself to work to collect money in order to furnish a home for the nurses of the Royal Infirmary, who, as the buildings of the institution afforded them no accommodation, have to live outside. A house was taken in Ravensworth Terrace, and this Mrs. Lord's committee have furnished. £203 was raised by subscriptions, and £180 by means of a ball. On the 3rd inst. Mrs. Lord had the pleasure of formally handing the gift over to the nurses, who entertained their visitors afterwards to tea.

### SHORT ITEMS.

There is little hope for candidates desiring admission into the Royal Hospital for Sick Children at Glasgow quickly entering it. There were 163 applicants for six vacancies on the last admission day.—Lord Salisbury has sent a donation of £100 towards the proposed County Home for Nurses in Dorset.—Coltishall has a small nursing home which stands as a refuge for the patients who are not suitable for the Norfolk and Norwich Hospital, and who are yet better away from their own homes. One nurse divides her time between the home and the district. Last year 23 patients passed through it, the larger proportion of whom were cured. The balance to the good after an expenditure of £161 is £26.——The Duchess of Sparta is reorganising the nursing in the Ecole Militaire—that is, the Military Hospital at Athens. She has decided to put the matter under the control of an English superintendent-sister and two English nurses, and, as we are informed, has asked Mrs. Bedford Fenwick to make the selection.

### Antiseptics for Nurses.

By a Medical Woman.

### II.—ORIGIN AND DEVELOPMENT OF THE ANTISEPTIC TREATMENT OF WOUNDS (*continued*).

In 1835 a French surgeon, Jules Guyot by name, made a number of experiments to determine what effect cold had on the healing of wounds, not being content to accept, without proper investigation, the theory that the cooling and drying effects of the air on a wound caused bad results. As an outcome of these experiments, Guyot was convinced that wounds healed most satisfactorily at a temperature of about 98° Fahr. Consequently in the treatment of all wounds, he endeavoured to maintain this temperature by enclosing the limb in a box with glass sides, into which air at the above temperature was introduced, and this was continued for something like a fortnight. The wound was not covered with any dressing, so the whole process of healing could be easily watched, and was seen to consist in a copious discharge of pus, which scabbed over the wound. Guyot himself considered he got very good results, but his methods were not followed by anyone else.

At this time, indeed for some years before, the ill-effects of the air, or at least of something contained in it, on wounds had been recognised, and several surgeons had adopted the subcutaneous method of making wounds, so as to exclude the air, but it was Jules Guérin who, in 1841, not only practised it, but also did much to popularise it by his writings.

Some sixteen years later our own countryman, William Adams, strongly advocated subcutaneous surgery, and thus, through the teachings of Guérin and Adams the principle was widely adopted, and led to further developments in the form of what was known as "occlusion," or the exclusion of air from a wound by first of all making the incision as small as possible, and then applying such things as diachylum plaster, gold-beaters' skin, caoutchouc, &c., to protect from the air. These simple applications were not, however, long considered satisfactory, since the discharge collected under them, gradually lifted them up so that both discharge and air came into contact with the wound, and decomposition was speedily set up. To avoid this Jules Guérin invented an apparatus which fitted close to the limb, and from which the air was pumped out, and at the same time all discharges removed from the wound; but the apparatus was clumsy, and apt not to work well, and moreover the results obtained being by no means satisfactory, it was given up.

The next method for excluding air was to cover all wounds with several layers of cotton wool, so as to filter the air, and render it harmless if it should happen to reach the wound. The further advantages claimed for this method were equal compression, equable temperature, lessened pain, freedom from traumatic fever, and either freedom from or greatly diminished suppuration. To secure these advantages the packet of wool must be opened and applied to the wound in an adjacent room, and not in the ward. Pasteur and others pointed out that as the wool was not sterilized it did not make an antiseptic dressing; but, nevertheless, some excellent results were obtained by its use.

Some interesting experiments were tried both in this country and in France on the effect on wounds of the different gases composing the air, which resulted in the discovery that carbonic acid gas was far more favourable to healing than any other. In a few instances wounds were kept in an atmosphere of carbonic acid gas, but this treatment had many disadvantages, and was very soon abandoned.

It is curious that whilst in England and France surgeons were devising means for excluding air from wounds, in Germany an exactly opposite, *i.e.*, the "open air," treatment was in vogue. There was no attempt at anything in the form of a dressing, and merely a piece of gauze was put over the wound to keep off flies, but, nevertheless, some very good results were recorded. The only precaution taken was to isolate the patient in a small room. In this country Professor Humphrey tried this method at Addenbrooke's Hospital and was an enthusiastic advocate of it. The next step was to encourage "healing under a scab," and the process was hastened by applying various powders, salicylic acid, zinc oxide, &c., to mix with the discharges from the wound, and make them dry up more quickly and form a scab. This method succeeded well in the case of small wounds, but completely failed with large ones. The same end was obtained by blowing air on the surface of the wound, and was called "ventilation of the wound." The air was blown on by means of an indiarubber bag for a quarter of an hour at a time, and repeated at least four times a day, until a sufficiently thick crust had formed. If any bad symptoms appeared the scab was removed, and any pent-up discharge removed, and the wound again "ventilated" to re-form the scab. Its originator, Bouisson, claimed, amongst other advantages, some antiseptic action for his method.

At one time, though not extensively, wounds were treated by cauterisation, either by actual cautery or preferably by chloride of zinc.

It was decidedly a great advance when Bennion, of Oswestry, introduced the treatment of wounds by covering them with lint soaked in an antiseptic solution, and, as might be expected, obtained excellent results.

We have hitherto omitted all mention of one important form of treatment, which should find a place in any account of antiseptics, and that is by water, either in the form of irrigation or baths. This treatment has been in vogue since the earliest times, but only since 1834 has much attention been paid to it, and first by Mayor, who obtained good results from it, and strongly advocated its use. Whenever practicable he placed the diseased part in a bath for several consecutive days, keeping the water at a temperature of 25 deg. C., and so arranging that the water could be renewed when necessary without removing the bath. This treatment had many advocates, but it never became general on account of the difficulties attending its application and the frequent accidents which occurred.

Although surgeons had applied antiseptics to wounds during several previous centuries, it was not till 1859 that general interest was awakened in this subject owing to the publication of a treatise by Corna and Derneaux. Pure alcohol was first used very extensively, in some cases combined with other substances, but it was always the active antiseptic principle. Of course other antiseptics came to the fore, but for the most part were found to possess some drawback which prevented their coming into general use. Such were glycerine, which was in great favour in 1855, and for a few years subsequently; and chlorate of potash, which proved too feeble an antiseptic for general use. Iodine also had its day, and was used at one time for painting the surface of wounds, and now survives in the shape of iodoform.

It was in 1815 that the antiseptic properties of coal-tar were first discovered, and it was used by various surgeons to purify wounds, both in this country and in France; but until Lister and other investigators took up the matter, antiseptic treatment did not rest on any rational or scientific basis, but was employed because in some way or another, then unknown, the discharges from wounds were greatly lessened, and the wound itself could be kept in a healthier and cleaner condition than when such antiseptics were not used. About 1864, Lister in this country and Lemaire in France began quite independently and unknown to each other to carry out investigations into the method in which antiseptics act in preventing putrefaction. Lemaire, however, did not follow up his investigations fully, whilst Lister published his results about 1867.

### Nursing in Paris Hospitals.

### A.—LAY NURSES.

### II.—The Campaign for their Introduction from 1878.

In 1878, Gambetta having smitten the dynastic coalition hip and thigh, swept MacMahon out of office, and installed Grévy as his *roi fainéant*, the time of Dr. Bourneville and his fellow laicisers in the Paris Municipal Council had come. The attack on the nursing Sisters was begun in earnest.

On December 1st, 1878, the first hospital changed from the Sisters to lay matrons. This was the so-called temporary hospital in the ancient and impressive old asylum for incurables in the Rue de Sèvres (near the Bon Marché), turned into a general hospital during the Franco-German war, and since named after the discoverer of auscultation, Dr. Laennec, whose Paris residence was across the way. The Sisters from the Rue de Sèvres were, however, transferred to the new Tenon Hospital, so it was not much of a victory.

The Municipal Council claimed to have found an obstructive King Log in the person of M. Mickel Möring, the director of the Parisian Assistance Publique, and on May 4th, 1880, a successor to him was found to play King Stork for the poor nuns in the person of M. Charles Quentin. Thereafter, the laicisation went merrily forward. On October 1st, 1880, laics took full charge of the ancient edifice on the hill behind the Jardin des Plantes, La Pitié Hospital. La Pitié has ever since been the centre of the laicisation campaign. Leaving aside the laicisation of asylums, I may note that the next to exile the Sisters (August 1st, 1880) was the great eastern foundation of the Revolution, Saint Antoine, whence nearly a century before the Cistercian nuns had been banished to found the hospital itself. The laicisation of Saint Antoine was followed (June 1st, 1882) by that of the women's Lourcine (now Broca) lock hospital. The same day the Tenon Hospital saw the lately-installed Sisters depart, although on March 8th of the year before the medical staff had protested against the contemplated change. Now came a short breathing-space in the secular crusade. The advocates of the nursing Sisters made a determined stand, and succeeded in ousting M. Quentin for administrative irregularities, he being replaced (November 4th, 1884) by M. Edouard Peyron, the present chief. The new director proved a more astute and determined laiciser than his predecessor. After many violent recriminatory discussions in the Council, on November 6th, 1885, by 51 to 10 votes, the laicisation of the Necker and Cochin Hospitals was demanded. On the 17th of this month a great part of the doctors and surgeons in every hospital in Paris issued a remarkable protest against this exile of the Sisters, a protest I shall in another place have occasion to refer to. On the 28th of this month, by 57 to 11, the Council passed a vote of regret that their demand had not been complied with.

M. Peyron, though a willing laiciser, was not to be caught in the irregular methods of his predecessor, who had neglected to consult the Council of the Assistance Publique. This mis-step had given a pretext for the vice-president and acting head of the Council, M. Henri Davillier, to resign in a sort of Parthian shaft letter to President Grévy. M. Davillier's successor, M. Emile Ferry, was, however, an ardent lay disciple, so in a few weeks (December 21st, 1885) that pious foundation of the southern tip of old Paris, the Cochin Hospital, was taken from the religious matrons. This occasioned a legal protest from the old founder's family, but in vain. The years 1886 and 1887 were each marked with three secularisations in the hospitals, *i.e.*, April 1st, 1886, the Foundling Hospital; October 28th, 1886, the Sick Children's Hospital, with the adjoining old Necker Hospital; May 1st, 1887, the old Foundling Hospital, near Saint Antoine (made a sick children's hospital in 1853, and named after the Empress, but renamed Trousseau in 1880); again, September 15th, 1887, the great democratic modern benevolence of Lariboisière, by the Gare du Nord; and on October 1st, 1887, the old aristocratic piety Beaujon, in the fashionable Faubourg St. Honoré.

During these two triumphant years of the lay campaign, 1886 and 1887, the Municipal Council had numerous discussions on the theme and emitted various votes. Thus, on March 19th, 1886, by 57 to 11, it felicitated M. Peyron on the progress of the work. On June 16th, 1887, it voted by 54 to 7 a peremptory demand for the complete expulsion of the remaining Sisters. Finally, on January 23rd, 1888, the last accomplished laicisation of any hospital in Paris itself took place. This was that of La Pitié's venerable sister State foundation, La Charité, in the artistic centre of the Seine. Various new hospitals had been organised during these years, all, of course, upon a lay basis, and in 1888 only the mother hospital, the Hôtel Dieu (and the largest hospital also venerable), the skin disease establishment of St. Louis, remained in the hands of the Sisters. For ten years the Sisters have held their own in these two last establishments, though continually threatened. In fact, way back in 1888, on September 1st, an order was issued by the Prefect of the Seine for the Sisters to depart from both hospitals on the 1st of the succeeding December. This order was the subject of an appeal at law, and the laicisers have been baulked for the present, in each case for two different reasons. The Hôtel Dieu is the mother-house of the Augustine Sisters of Charity, while St. Louis was given to the Sisters by special decree of Napoleon, and only another special decree of President Faure, or some other successor of Napoleon as Chief of the State, can expel them. Not only have the Hôtel Dieu and Saint Louis not been laicised, but during the last few months a third hospital with the Sisters has been added to the establishment of the Assistance Publique. This is the magnificent benefaction of the late Madame Boucicaut of the Bon Marché, a remarkable foundation I shall have occasion to particularly refer to.

During this long campaign, the succeeding Ministers and the National Legislature have occasionally taken an indirect hand in the struggle. In the Senate, on May 31st, 1881, M. Lambert de Sainte Croix succeeded in carrying a resolution in favour of the Sisters by 135 votes to 120, the previous question, moved by M. Scheurer-Kestner, being defeated by 111 to 139. The debate was signalised by an unexpected announcement by the Minister of the Interior (M. Constans) that the

Sisters of Saint Martha had themselves asked to be retired from La Pitié, and had threatened to leave St. Antoine unsupplied, their order being so very much weakened. This elicited a sharp retort from M. Buffet, who said the laicisers would wait long before the other orders of nursing Sisters lacked recruits. M. Buffet quoted an eloquent tribute to the Sisters of Charity delivered in 1850 by Victor Hugo. Nevertheless, the Victor Hugo of 1881, who sat in the Luxembourg as an octogenarian Senator, voted with the minority against the Sisters in both ballots. In the Senate, again, on May 30th, 1883, M. Bérenger brought up the question of Sisters in connection with the subject of hospital chaplains. The Minister of the Interior (now M. Waldeck-Rousseau) by a small timely concession avoided an adverse vote. On a third occasion, December 19th, 1885, M. Duprès brought before the Senate the imminent laicisation of the Cochin hospital. The Minister of the Interior (now M. Allain-Targé) claimed that the Seize Mai was responsible for the whole lay campaign. Like his predecessor, M. Allain-Targé entrenched himself behind the absolute legal right of the Paris administration to conduct the internal affairs of the hospitals to suit itself. The Senate passed by the matter without a vote. In the Chamber of Deputies the subject has constantly cropped up in the shape of questions. Once the late Dr. Armand Duprès, then a member of the Chamber, as well as a surgeon of La Charité and a fierce opponent of laicisation, brought on the matter in a formal way on June 21st, 1890. His motion was adjourned by a vote of 248 to 231, and it was not until December 20th following that he succeeded in obtaining a discussion, which occasioned a remarkable discourse on the legal aspect of the case by M. Constans, a discourse I will have reason again to refer to. M. Duprès was defeated by a vote of 356 to 176. The chief public notices of the question since 1890 have been acrimonious discussions in the Municipal Council over the cost of laicisation in the budget of the Assistance Publique, and some votes on the Hôtel Dieu and Saint Louis, the last formal demand for their laicisation being voted a few weeks ago. In the sitting of the Council, October 31st, 1892, M. Alpy moved for a reinstatement of the Sisters, being defeated by 54 to 13 votes. In the same sitting M. Quentin-Bauchat moved that a hospital be organised, under the charge of the Sisters, on each bank of the Seine for such patients as preferred religious nursing. This was also defeated by 48 to 13 votes. A resolution for the laicisation of the two remaining hospitals was then moved by M. Louis Lucipia, and adopted, being a reiteration of a vote carried by 54 to 11 on October 24th, 1890.

Although no laicisation has taken place in any hospital in Paris from 1888 to 1898, in 1894 the Sisters were discharged from the city's hospital for scrofulous children at Berck-sur-Mer, though they still remain in the children's convalescent hospital at La Roche Guyon, in the Seine et Oise, in compliance with the founder's will.

E. R. Spearman.

### Religious Influences in Nursing.

Every now and again the question of different religious creeds makes itself felt in institutions employing nurses. It is easy enough to raise the cry of bigotry and attempt to level all down to the toleration of indifferentism, but the course has not led to any very satisfactory result. Deep and sincere religious motives are acknowledged by all to be a most important factor in the making of a good nurse, and the trouble only begins when the forms in which these motives are enshrined vary. No one doubts that every creed has its saints, but it is frequently forgotten that all its disciples have not yet reached that spiritual elevation whence they can overlook differences of form and rejoice in unity of spirit. Certain concessions are due to the weaker brothers and sisters, and it is good to bear in mind that the weaker members of one period may be the strong ones of the next. Many of those who have the management of large institutions would feel deeply thankful if they had only to consider the religious requirements of one creed. Personally they may care little whether the creed be Roman Catholic, Church of England, or Nonconformist of any one special sect, provided that all the nurses under their control held the same faith, but woe betide the matron who endeavours to eliminate the discordant element of varying creeds from the ranks of the nurses. Whether she were an angel or an atheist she must face an outcry that might well make her wish to wash her hands of the whole concern. The one thing that incenses the public at the present moment is tenacity of religious purpose; in fact the popular creed is that you may believe what you like, but must keep it out of sight, and must ask no one else to join your faith.

There are some who regard nursing as a means of propagating a special creed. But the duties of a nurse are to wait upon the sick, not to teach a religion, and she will further the cause of her faith more by her example and the zeal with which she performs her appointed task than by any number of words.

The point now in our minds is the desirability or non-desirability of uniformity of creed in each nursing residential home or society, and whether the constitution of a guild, such as the Church Nurses' Guild recently formed for nurses who are members of the Church of England, is a step in the right direction or not. In thinking over the *pros* and *cons* relating to such guilds, their formation appears to be due to three causes apart from any taint of bigotry. The first is the known deterioration in the character of nurses not actuated by religious motives; second, the gain of strength accruing to the individual by living with others who are in accord with her on such subjects; and, third, the absence of friction when the needs of one and not many creeds have to be considered and provided for.

On the other hand, it is almost impossible to feel strongly upon any subject whatsoever without wishing to impress one's own conclusions upon others, and as the strongest feelings of the creature naturally centre round the Creator, this disposition is intensified in all religious questions. Living in the world, where the individual is perforce jostled against others holding every shade of opinion on every conceivable subject, rubs off unnecessary idiosyncrasies and teaches a wider charity. The simile of the pebbles on the shore rounded by the friction against one another caused by the moving waters is well known and most true.

It will be seen from this statement of the case that in the nursing world both the tenacious religious principle and the toleration of other people's views which friction teaches are necessary, and so long as guilds help the first without hindering the second they are beneficial; but once let a guild foster bigotry in any shape or form, the day of its usefulness is over.

We live in an age of co-operations, and it seems to us that a wise use of these associations is most helpful to the individual. If, then, nurses join religious co-operations which help the growth of their spiritual natures, and at the same time remember that these are not infallible, the religious guild will take its rightful place in the social economy and counterbalance the lack of religious stability that is, unfortunately, only too prevalent.

### The Book World for Women and Nurses.

[We invite Correspondence, Criticism, Enquiries, and Notes on Books likely to interest Women and Nurses. Address, Editor, THE HOSPITAL (Nurses' Book World), 28 & 29, Southampton Street, Strand, London, W.C.]

THE NURSE'S HANDBOOK OF COOKERY. By Miss E. M. WORSNOP, assisted by Miss M. C. BLAIR. (London: Adam and Charles Black. 103 pages. Price 1s. 6d.)

It would be difficult to praise too highly this excellent book, which will indeed be "a help in sickness and convalescence," not only to the trained nurse, but also in every household. Few houses are free from the experience of having an invalid who may require special diet for a time, or an appetite only tempted with something outside the ordinary household fare. The introduction to Miss Worsnop's book is written by Miss Emily Briggs. This, in itself, is a guarantee of the worth of the book. In it she mentions that for sixteen years Miss Worsnop has been an enthusiastic and successful teacher in one of the centres of the London School Board, and is thoroughly qualified to produce a book of this kind. The leading chapters on various foods are full of interesting details, which every nurse would do well to know. Even if she is not in the position to arrange for the food of the patients, it will help her to know the why and wherefore such food is ordered. Miss Worsnop explains all so clearly, and keeps to the scientific principle of rendering food as digestible as possible. The quantities required are plainly stated at the head of every recipe, followed by instructions how to use them; these are so clearly put, and every detail added, that there is little or no room for failure if carried out conscientiously. Miss Worsnop has thought of food for every stage of illness—from the weak patient who can only take peptonised foods, beef teas and broths, to the convalescent who enjoys a "well-grilled chop" and "marple pudding." Each recipe has been repeatedly tried. Thus, with sure confidence of the success with which each dish will turn out, we can recommend this book to every nurse and household.

THE PROPHET'S MANTLE. By CHRISTABEL R. COLERIDGE. (Isbister and Co. 1897.)

Early one morning Mark Gladwyn, the newly-ordained curate of Ashdew, was called up to marry a couple by special licence. They gave their names as Norman Gerard Wentworth and Helena Winifred Wynne. It was in January, a snowy morning, and the church was dark. So the curate's brother, George, who had followed him out of curiosity, held a candle so that its light fell on the Prayer-book. The couple were accompanied by two friends, an elderly gentleman and lady, who acted as witnesses. In the vestry the register was duly signed, the ages of the bride and bridegroom being given as 18 and 21. "The young man (the bridegroom) turned like an automaton, and offered his arm to his bride. She took it, and they walked down the church, and, as far as George could see, never spoke to each other." Then the four strangers got into the fly which had brought them, and drove away. "I say, Mark," said George; "you'll hear of this again, I bet! Very queer customers, indeed! That chap was no more twenty-one than I am, and I don't believe the bride was grown up." And then the curate did a rather unusual thing. He sent his fee back to the address which the bridegroom had given him, and it was returned from the Dead Letter Office with "Not known here" scrawled across it. Soon after this George Gladwyn was sent to the private school of the Rev. Mr. Fleming, of Longhurst Rectory. When he had been there about three months a new pupil, Norman by name, arrived. The schoolfellows were introduced. "George gave a great start and fell suddenly silent. His sharp eyes had recognised Norman Gerard Wentworth, the hero of the mysterious marriage at which he had so recently assisted." Norman or Wentworth did not recognise him. "The oddest part of it was that there was nothing odd about Norman. He had come from a public school, and had brought the most proper tastes and behaviour with him. He played golf and tennis, did his work with intelligence, but without enthusiasm, never fell into gloomy silence, or behaved in any way becoming the possessor of a guilty secret." How Norman went abroad and disappeared; how George Gladwyn afterwards met the mysterious bride, and fell in love with her; what complications ensued; what struggles of conscience Gladwyn endured; what difficulties Helena, who had parted from her husband at the church door, encountered; what it all meant; and how it all ended, the reader must find out for himself. The story is well written and, on the whole, interesting, without being in the least sensational.

### Too Clever by Half.

THE affairs of the Royal British Nurses' Association must always be of interest to nurses, which must be our excuse for mentioning a suggestion in the *Nursing Record* which, if accepted as true, or if imagined to be even founded on fact, might lead to erroneous conclusions. It appears that Mr. G. B. Hudson, M.P. for the Hitchin Division, Herts, wrote a letter to the *Lancet* inquiring whether the Incorporated Medical Practitioners' Association was entitled to speak authoritatively in the name of the medical profession. This letter was reprinted in the *Nursing Record* with the following characteristic little note: "This ingenuous document has a strangely familiar ring to us. We ventured, therefore, to look into the 'Medical Directory' for some solution to the mystery, and found that a gentleman of the name of Charles Elliott Leopold Barton Hudson is assistant surgeon to the Middlesex Hospital; but, of course, it is a mere flight of imagination to suppose that he is any connection whatever of Mr. G. B. Hudson, M.P. for Hitchin." Of course, everyone will see the sneer and the suggestion. The Middlesex Hospital (containing as it does Mr. Fardon) is like a red rag to the editor of the *Nursing Record*, and great, no doubt, was her joy when she thought she had traced this letter to the Middlesex. But unfortunately for her she looked in an old Directory. Had she looked into this year's Directory she would have found that Mr. C. E. L. B. Hudson, who was well-known in medical circles, died nearly a year ago, and therefore can hardly be accused of prompting Mr. Hudson, M.P., who, it should also be said, is no relative.

### Marriage.

A MARRIAGE of somewhat unusual interest was celebrated in Melbourne last November. The bridegroom, Mr. James S. Buchanan, is M.B., F.R.C.S., and his bride, Miss Alfreda Hilda Gamble, is also M.B. The lady, after a successful career at the University of Melbourne, came out second in the list of the six most distinguished students at the final examination. She has since spent a year in hospital work, and is now engaged in private practice. She and her husband will continue their work together.

### Minor Appointments.

HACKNEY UNION INFIRMARY.—Miss Martha West, who was trained at St. Mary Abbotts Infirmary, Kensington, and who was subsequently staff nurse at the Lewisham Union Infirmary, was appointed Charge Nurse of this infirmary on February 2nd.

### Everybody's Opinion.

[Correspondence on all subjects is invited, but we cannot in any way be responsible for the opinions expressed by our correspondents. No communication can be entertained if the name and address of the correspondent is not given, as a guarantee of good faith but not necessarily for publication, or unless one side of the paper only is written on.]

### NURSE-ATTENDANT.

A "NURSES' WELL WISHER" writes: I regret to read in your journal the unfortunate experience of "An Invalid" respecting sick or trained nurses. During a serious illness lasting nearly three years, I have been much more fortunate. Perhaps, as this is the case, it is only just to the nurses to give my experience. The trained nurse who is at present with me has been nursing me for some time, and of her sympathy, patience, and skill I cannot speak too highly. At intervals I have had three other nurses from an institution in Surrey, and each one in her faithful discharge of duty has far exceeded a hireling's service. I may add that a month's trial of a nurse-attendant only made me more highly prize the skilful touch and more practised eye of the trained nurse. Probably your correspondent has been unfortunate in her choice of institute.

### "ERGOT" AND "CERTIFICATES."

MRS. ELINOR AGNES BEDINGFELD writes: Seeing your reply to "Nurse P." (149) in present number of THE HOSPITAL, I beg to state that during the nineteen years I have been in practice as a midwife I have invariably, in cases in which no doctor has been in attendance, given the undertaker a certificate in the case of stillborn children, wording it as follows: "I hereby certify that I delivered Mrs............of............of a stillborn male (or female) child on the ............ day of ............ 189.... Signed ............ Certified from..........Hospital and L.O.S." The undertaker has always deemed this sufficient. No doctor could certify to stillbirth unless he was present at it, so if the midwife's word was not taken an inquest would have to be held in every case of stillbirth that was attended by a midwife only. In reply to "One in Suspense," I would advise her to go in for the examination held by the London Obstetrical Society. She can do this without training in a lying-in hospital if she can bring proof that she has delivered twenty-five cases to the satisfaction of a qualified medical man, and that she has attended lectures on midwifery. If she passes this examination she will be a certificated midwife, and can then give ergot if necessary. Chemists will not as a rule sell ergot to unqualified midwives, as it comes under the Poisons Act. A patient might lose her life in certain cases if a midwife waited for a doctor to administer ergot. I give it as a matter of routine after every labour, and during labour if I think it is needed. If midwives are not to give it without a doctor what is to be done in country districts, such as I have worked in, where there was no doctor nearer than four miles, if one has a severe case of post-partum hæmorrhage?

*** A midwife has a perfect legal right to give or persuade a person to take a harmless dose of ergot, but she has no more right to do so than anyone else, and the certicate of the L O.S. gives no greater right than that given by St. Mary's Hospital, Manchester. In regard to the certificate of stillbirth, a midwife gives it merely as "a person present at the birth." The law gives the midwife no special status.

### THE TRIALS OF A MONTHLY NURSE.

MRS. ELINOR AGNES BEDINGFELD writes: "Dora H." should have a clear understanding with her committee as to the number of hours she is to work per diem. Eight is the usual number, except in emergencies. I am not allowed to do night work except in midwifery cases, assistance being given, by the committee's leave, in cases needing night attention. The doctors "Dora H." works for are certainly inconsiderate. I remain with a case for from one and a half to two hours after delivery, supposing all to be going on well, leaving word where I am to be found if wanted. I pay a second visit in the course of a few hours. If the doctors require their patients waited with so long they should provide another nurse. I work principally as a midwife, and sometimes have two or more confinements in the twenty-four hours, so could not stay with my patients for long unless there was an urgent necessity. In answer to "A Perplexed Monthly Nurse and L.O.S.," I can say that a monthly nurse does not get regular "off-time" as other nurses do. If there is a good children's nurse in the house, she may, after the first week, perhaps, take charge of the sick room while the nurse runs out for half an hour, but as a rule the monthly nurse does not get out till the baby can be taken out too. It is then her duty, when the weather is fit, to take the child out every day, and so she gets fresh air herself. "F. Taylor," if she intends to continue nursing, should take to heart words of Canon Newbolt's, "It is a glorious thing to be allowed to assist in repairing the temple of the Holy Ghost." I fear she has mistaken her vocation. Husband-hunting and nursing cannot be done together.

### Appointments.

### MATRONS.

ST. CROSS HOSPITAL, RUGBY.—Miss Caroline Walker was appointed Matron of this hospital on the 14th inst. She was trained at the Victoria Hospital for Children, Chelsea, and at the London Hospital. Miss Walker was afterwards sister at the Colonial Hospital, Gibraltar, and at the Government Civil Hospital, Hong Kong, for the last five years.

MILLERTON INFECTIOUS DISEASES HOSPITAL, BERWICKSHIRE.—Miss Gale, who was trained at the Workhouse Hospital, Liverpool, was appointed Nurse-Matron here on the 9th inst. She has been engaged in private nursing in Newcastle, and more recently as charge nurse of the Fever Hospital, Kilwining.

BRISTOL ROYAL INFIRMARY.—Miss A. B. Baillie was appointed Matron of this infirmary on the 8th inst. She was trained at the London Hospital and was afterwards sister of that hospital for four years, when she became matron of the Rugby Hospital of St. Cross.

NETLEY.—Miss Alex. Guthrie has been appointed Sister at the Netley Soldiers' Hospital. She received her training at Cardiff Infirmary and Queen Charlotte's Lying-in-Hospital, and afterwards held the position of sister at Cardiff Infirmary.

BRIDGEND COTTAGE HOSPITAL.—Miss Fanny Smithies has been appointed Matron at this institution. She was trained at Bolton Infirmary, and was for four years surgical sister at Cardiff Infirmary.

IN announcing Mrs. E. Bowen's appointment as Matron to the new Isolation Hospital, Worcester, last week she was erroneously described as "Miss."

### Wants and Workers.

[THE attention of correspondents is directed to the fact that "Helps in Sickness and to Health" (Scientific Press, 28 & 29, Southampton Street, Strand, London, W.C.) will enable them promptly to find the most suitable accommodation for difficult or special cases.]

MISS E. A. HANCOCK, 156, Anerley Road, would be glad to have a second-hand copy of Burdett's "Hospitals and Charities" for 1897.

MISS SCHNEIDER, Freio Strasse, 34, Zurich, will be glad to receive back copies of the "Nursing Mirror," and will pay the carriage if any nurse having them at her disposal will write to her.

### For Reading to the Sick.

LENT.

I ACKNOWLEDGE my faults, and my sin is ever before me.—*Psalm* li. 3.

**Verses.**

Thou the shame, the grief hast known;
Though the sins were not Thine own,
Thou hast deigned their load to bear,
Jesus, Son of Mary, hear!

Once more the solemn season calls
A holy fast to keep;
And now within the temple walls
Let priest and people weep.

But vain all outward sign of grief,
And vain the form of prayer,
Unless the heart implore relief,
And penitence be there.

We smite the breast, we weep in vain,
In vain in ashes mourn,
Unless with penitential pain
The smitten soul be torn.

—*Hymns Ancient and Modern.*

Forty days and forty nights,
Thou was fasting in the wild;
Forty days and forty nights
Tempted and yet undefiled.

Sunbeams scorching all the day;
Chilly dewdrops nightly shed;
Prowling beasts about Thy way;
Stones Thy pillow; earth Thy bed.

Shall we not Thy sorrow share,
And from earthly joys abstain,
Fasting with increasing prayer,
Glad with Thee to suffer pain?

Keep, O keep us, Saviour dear,
Ever constant to Thy side;
That with Thee we may appear
At th' eternal Eastertide.

—*Hymns Ancient and Modern.*

Prostrate your soul in penitential prayer,
Humble your heart beneath the mighty hand
Of God, Whose gracious guidance oft shall lead
Through sin and crime the changed and melted heart,
To sweet repentance and the sense of Him.

—*Clough.*

**Reading.**

True repentance must reduce to act all its holy purposes. A holy life is the only perfection of repentance.—*Jeremy Taylor.*

Of all acts, is not for man repentance the most divine?—*Carlyle.*

"Jesus said unto them, Come ye yourselves apart unto a desert place, and rest awhile."

I have now begun the holy season of Lent. It is a time to spend with God. At other times it is my duty to think chiefly about my work; but in Lent it is my duty to think only as much as is necessary about my work, and to think very much about my own soul. Am I reconciled with God? If I die will He be able to own me, or will Satan claim me? These are the questions I have to answer. Nobody ever yet spent a good Lent who was not better at the end than he was at the beginning. I cannot think about God and not get to love Him. He is so good and great and wonderful, and the more He is in my thoughts the more I shall admire and love Him. Then, when Easter comes, I shall have become more like God and more fit to live with Him in Heaven.—"*A Lent with Jesus.*"

### Notes and Queries.

*Probationer.*

(160) A young lady is anxious for a place as probationer. We have written most of the hospitals, &c., around here, and find there are no vacancies. Have you a directory of hospitals?—*R. D.*

See Honnor Morten's "How to Become a Nurse," published by Scientific Press, 28 & 29, Southampton Street, Strand. See also Burdett's "Hospitals and Charities," or "Burdett's Official Nursing Directory," same publishers.

*Free Massage.*

(161) Is there any place where a poor woman could have massage free?—*Janet.*

Apply to the Society for Trained Masseuses, 12, Buckingham Street, Strand.

*The Cost of Food.*

(162) Will anyone who is experienced in convalescent home work kindly tell me what ought to be the average cost of each patient per week for food alone, ordinary diet, in a small home of, say, 10 people—five men and five women?—*Matron.*

About 7s. 6d. a head per week is a fair average.

*Country Homes for Children.*

(163) Can anyone give a list of convalescent homes for poor children? I am nurse in a London parish, and am often asked to get children into country and seaside homes.—*Sister Nono.*

Children's Home Hospital, 1, Park Road, Barnet; terms 5s. a week. Lady Brassey's Home, 3, Cantelupe Street, Bexhill-on-Sea, for boys 4-10, girls 4-14; payment 5s. a week. Charlotte Children's Convalescent Home, 19, Westbourne Villas, Brighton; free. Children's Country Holidays Fund, 10, Buckingham Street, Strand. See Burdett's "Hospitals and Charities" for others.

*A Nurse's Equipment.*

(164) Is it usual for a district nurse to provide the bag and its fittings? Is she expected to stock powders, ointments, antiseptics, and dressings?—*E. D.*

A nurse provides her own bag, her committee the dressings, &c.

*Homes of Rest in the North of England.*

(165) A matron would be glad to know the names of any homes of rest in the North of England suitable for a phthisical female patient.—*H. J. M.*

A Home of Peace, Sunnyside, Ashton Old Road, Openshaw, Manchester. Southport Convalescent Hospital and Sea-Bathing Infirmary. Convalescent Hospital, Silloth. Cumberland and Westmoreland Convalescent Institution, Silloth. The Evans Convalescent Home for Children and Women, Solihul, Birmingham. Seaside Home for Sick Women and Children, Whitby. See Bardett's "Hospitals and Charities" for others, published by the Scientific Press, 28 and 29, Southampton Street, Strand.

*Bedridden.*

(166) Can anyone tell Nurse Copley of a home for incurables for a bedridden woman?

If this is not a case suitable for the infirmary consult guide to the local charities in your neighbourhood. If there is not one see Burdett's "Hospitals and Charities," the Scientific Press, Southampton Street, Strand, London, W.C.

*Medico-Psychological Certificate.*

(167) Can a nurse obtain the certificate of the Medico-Psychological without going into an asylum?—*Nurse.*

Three years' work in an asylum is essential before a candidate may try for this certificate. But as this time may be divided between two asylums "Nurse" may enter another for a year. The secretary of the Medico-Psychological Association, 11, Chandos Street, W., will give full particulars.

*Diseases of the Nerves.*

(168) Will you kindly tell me the name of a small book on "diseases of the nerves" in the cheapest edition?—*Daisy.*

This is far too wide a subject to be treated in a small book.

*Goitre.*

(169) Can you tell me if there are any special books published on goitre and its treatment? Also, if any lectures on the same subject have appeared within the last year in any of the medical papers.—*Consultation.*

Read the article on goitre in a good recent encyclopædia, or in Quain's Dictionary of Medicine. Articles constantly appear in the medical journals.

*Paralysed Patient.*

(170) Can you kindly tell me of a home where a paralysed lady could be received by paying £1 or £1 1s. per week, near Worthing or south or south-east of England preferred?—*Nora.*

The Royal Hospital for Incurables (office, 106, Queen Victoria Street, E.C.) Seaside Home is at St. Leonards; Home for Invalid Ladies, St. Mary's, Dean Park, Bournemouth; £1 1s. a week. St. Bernard's Invalid Gentlewoman's Home, Brighton; payment 15s.-30s. a week. Convalescent Institution for Invalid Ladies, Erith House, Torquay; 15s.-£1 2s. The Richmond Hospital and Home, Worthing, 14s. 6d. weekly.

*Canary Islands.*

(171) Will you let me know are there vacancies for nurses in Canary Islands? Or would it be a likely place for nurses to get up a connection? Also what prospect is there for pharmaceutical chemists? 2. Is there any lying-in hospital in Manchester where a certificate could be obtained for midwifery in three months? If so, what would be amount of fee?—*E. Ster.*

Write to the Secretary, the Colonial Nursing Association, the Imperial Institute, W., for nursing prospects in the Canary Islands. 2. The Maternity Hospital, Manchester, trains midwives for three months; fees £15 15s., and £5 5s. for lectures and certificate.

